# Room‐Temperature Molding of Complex‐Shaped Transparent Fused Silica Lenses

**DOI:** 10.1002/advs.202304756

**Published:** 2023-10-23

**Authors:** Ya Xu, Xiaotong Du, Zhenhua Wang, Hua Liu, Peng Huang, Suet To, LiMin Zhu, Zhiwei Zhu

**Affiliations:** ^1^ School of Mechanical Engineering Nanjing University of Science and Technology Nanjing Jiangsu 210094 China; ^2^ Key Laboratory for UV Emitting Materials and Technology of Ministry of Education Northeast Normal University 5268 Renmin Street Changchun 130024 China; ^3^ State Key Laboratory of Ultra‐precision Machining Technology Department of Industrial and Systems Engineering The Hong Kong Polytechnic University 11 Yuk Choi Rd Kowloon Hong Kong SAR 999077 China; ^4^ State Key Laboratory of Mechanical System and Vibration School of Mechanical Engineering Shanghai Jiao Tong University Shanghai 200240 China

**Keywords:** fused silica lenses, mass production, organic‐free, room‐temperature molding

## Abstract

The high hardness, brittleness, and thermal resistance impose significant challenges in the scalable manufacturing of fused silica lenses, which are widely used in numerous applications. Taking advantage of the nanocomposites by stirring silica nanopowders with photocurable resins, the newly emerged low‐temperature pre‐shaping technique provides a paradigm shift in fabricating transparent fused silica components. However, preparing the silica slurry and carefully evaporating the organics may significantly increase the process complexity and decrease the manufacturing efficiency for the nanocomposite‐based technique. By directly pressing pure silica nanopowders against the complex‐shaped metal molds in minutes, this work reports an entirely different room‐temperature molding method capable of mass replication of complex‐shaped silica lenses without organic additives. After sintering the replicated lenses, fully transparent fused silica lenses with spherical, arrayed, and freeform patterns are generated with nanometric surface roughness and well‐reserved mold shapes, demonstrating a scalable and cost‐effective route surpassing the current techniques for the manufacturing of high‐quality fused silica lenses.

## Graphical abstract

A room‐temperature molding technique is proposed to directly derive the complex‐shaped transparent fused silica optics from pure silica nanopowders without introducing organic additives. Miniature lens array, micro‐structured surface with sharp edges, and continuous freeform surface are demonstrated with nanometric surface roughness and well‐reserved mold shapes. The one‐cycle molding process lasts two minutes, providing a promising route for the industrial‐scale production of fused silica optics.

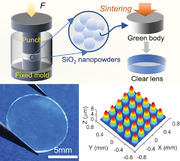

## 1 Introduction

Owing to the high transmittance over the ultraviolet, visible, and near‐infrared spectrum, fused silica glass with high mechanical, thermal, and chemical performance is outperforming, which has been widely applied as an excellent optical material for optical lenses in the industry.^[^ ^1^, ^2^, ^3^ ^]^ Although the high mechanical performance enables irreplaceable applications in severe conditions, it imposes significant challenges in manufacturing. To deal with the high brittleness and hardness, abrasive machining, including grinding and polishing, was employed as a predominant mechanical machining method to generate fused silica optics.^[^ ^4^ ^]^ Nonetheless, abrasive machining is commonly limited to process small batches of components with simple shapes, i.e., spherical and aspherical lenses with axis symmetry. Chemical etching, using polymer masks for structure protection and hazardous mediums for material removal, is commonly employed for structuring the fused silica at the micro or nanoscales.^[^ ^5^, ^6^ ^]^ However, it is challenging for the time‐consuming etching to flexibly control the real 3D surface shapes, i.e., freeform, and micro‐structured optical surfaces, etc.^[^ ^7^ ^]^ Alternatively, the high‐energy beam‐based direct writing technique employs lasers, focused ion, or electron beams to blast the desired complex shapes on fused silica glasses.^[^ ^2^, ^8^ ^]^ However, the essential point‐to‐point material removal makes it inapplicable for industrial‐scale optics productions.

By pressing the glass precursor on the molds at a temperature slightly above its glass transition temperature (*T*_g_), the glass molding technique is widely applied for the mass replication of optical lenses with even complex‐shaped freeform or micro‐structured optical surfaces.^[^ ^9^, ^10^ ^]^ To process the fused silica glass with a high *T*_g_ over 1400 °C, the mold produced from the glassy carbon or graphite is almost the irreplaceable choice in the industry to withstand such a high temperature.^[^ ^11^, ^12^, ^13^ ^]^ However, the highly rough surface and high wear rate inherently induced by the carbon material structure prohibit its application in generating optical surfaces.^[^ ^14^ ^]^ Therefore, it is nearly impossible to directly replicate the fused silica optics by glass molding to date. To circumvent the high‐temperature processing, the low‐temperature pre‐shaping strategy was recently developed through the liquid sol‐gel or silica nanocomposite approaches.^[^ ^15^, ^16^ ^]^ For the sol‐gel approach, the deliberately optimized silica network‐forming chemical solutions are additively manufactured to desired shapes by stereolithography^[^ ^17^, ^18^ ^]^ or direct ink writing.^[^ ^19^, ^20^, ^21^ ^]^ After slowly drying the shaped components, the high‐temperature debinding is conducted to remove the organics, followed by high‐temperature sintering to achieve fully densified fused silica glasses without crystallization.^[^ ^17^, ^18^, ^19^, ^20^, ^21^ ^]^ Since the strength of the cured wet gel objects is relatively low, the drying process usually requires several days to avoid ununiform shrinkages and cracks. Furthermore, the low strength makes it challenging to withstand the internal stress generated in drying, resulting in a structure warpage that is hard to control.^[^ ^22^ ^]^

For the silica nanocomposite approach, the silica nanopowders are stirred with the photocurable monomers, forming the liquid glass for fast facile replications^[^ ^23^, ^24^, ^25^, ^26^ ^]^ or fast prototyping^[^ ^1^, ^27^, ^28^, ^29^ ^]^ at room temperature. An alternative approach is to shape the polymerized silica nanocomposites through low‐temperature polymer‐based processing methods, for example, hot embossing,^[^ ^14^ ^]^ cutting,^[^ ^30^ ^]^ and injection molding,^[^ ^31^ ^]^ etc. Similar to the sol‐gel method, the high‐temperature debinding and sintering are essentially required to achieve the fully transparent fused silica components after pre‐shaping the silica nanocomposites at room temperature. Although slow drying is eliminated, a slow heating rate is commonly adopted for the debinding to evenly evaporate the organics to avoid structural collapse by rapid gas generation, which may significantly elongate the manufacturing time.^[^ ^23^, ^24^, ^32^ ^]^

An effective way to speed up the debinding in the nanocomposite approach was to minimize the organics in the pre‐shaped components.^[^ ^33^, ^34^ ^]^ For example, by adopting a specially designed solvent, the photocurable resins in the photopolymerized silica components were washed out with toluene at room temperature. For the washed components with limited organics, the debinding and sintering speed was increased by around five times.^[^ ^35^ ^]^ Furthermore, to lower the sintering temperature, a solvent‐free, pre‐condensed liquid silica resin^[^ ^36^, ^37^ ^]^ and a polyhedral oligomeric silsesquioxanes (POSS) resin^[^ ^38^ ^]^ capable of forming continuous silicon‐oxygen molecular networks were developed, which can be derived transparent glasses with a temperature reduction of 500 ^o^C as compared with that required by the dominant sol‐gel and nanocomposite approaches. No matter which approach is adopted for the process improvement, the organics are indispensable for forming the photocurable slurry for the pre‐shaping. In this situation, the slurry uniformity and the organic removal rate are crucial for achieving even stress distributions and high optical transmittance for the densified glass, requiring laborious stirring for the slurry homogenization and careful heating control for evaporating the organics. Therefore, although the photocurable silica slurry‐based approach provides a paradigm shift in fast replicating fused silica components, the inevitable stages of adding and removing the organics in the slurry will increase the operation complexity and process uncertainty for guaranteeing the generation of high‐quality fused silica lenses.

In this work, a room‐temperature molding technique is proposed to directly derive the complex‐shaped transparent fused silica optics from pure silica nanopowders without introducing any organic additives. Completely different from the photocurable silica slurry‐based pre‐shaping methods as reported, our approach adopts high pressure on the silica nanopowders against a complex‐shaped mold, thereby solidifying the nanopowders with the replicated mold shapes in minutes through the interfacial bridge connections. After densifying the compact lenses by atmospheric sintering, fully transparent fused silica lenses can be achieved with reserved molded shapes. Without organic additives, the tedious slurry preparation and high‐temperature debinding processes can be well avoided, which may significantly simplify the process and enable the industrial‐scale mass production.

## 2 Results and Discussion

### 2.1 Room‐Temperature Molding

**Figure 1:**
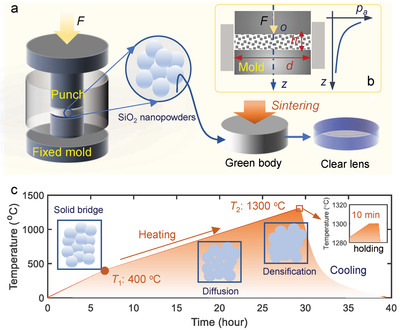
Schematic of the room‐temperature molding process, a) the basic process involving the axial press with a force *F* on the punch against a fixed mold with desired shapes, the silica nanopowders pressed to be the solidified green body between the two molds, and the high‐temperature sintering to derive the dense clear lens with replicated shapes, b) the pressure distribution along the axial direction induced by the side‐wall frictions, and c) the heating treatment in the sintering involving the two‐stage heating with different heating rates, the dwelling stage at the high temperature (*T*_2_), and the natural cooling down stage. The interface diffusion between the adjacent nanopowders occurs in the sintering and finally reaches the fully dense structure.

The schematic of the room‐temperature molding is illustrated in **Figure** **1**a, which mainly consists of the room‐temperature axial pressing stage and the atmospheric sintering stage. For the pressing, a metal mold with desired surface shape is fixed as the lower die, and a moving punch is adopted as the upper die. Constrained by the side walls, the silica nanopowders with an average diameter of ≈40 nm may uniformly fill the pressing space. Using a specified pressure provided by a hydraulic compaction press, the nanopowders are compacted through solid bridge connections between adjacent powders, forming a compact green body with precisely replicated mold shapes. In the pressing of nanopowders, nanopowder rearrangements, plastic deformations at the powder interfaces, and nanopowder fragmentations occurred sequentially to form a solidified compact.^[^ ^39^ ^]^ At the initial stage, the bridge connections were sufficiently low to allow the rearrangement of the nanopowders for randomly filling large pores in the pressing space. With the further movement of the upper punch, the pressure increased, and the pores in the random close packing state gradually became isolated and closed. When the bridging stress increased to be greater than the yield strength of the nanopowders, plastic deformations at their interfaces and local fragmentations occurred to form new active surfaces, which further improved the strength and density of the compact to withstand external crush forces.

The molding process lasts 2 min for each cycle, including the loading, holding, unloading, and ejection stages, enabling high rates for large quantity production. Due to the friction between the side walls and nanopowders, the internal pressure may gradually decrease to form a pressure gradient along the axial direction as illustrated in Figure 1b. As discussed in the Supporting Information, the nonuniform pressures may increase the warpage and irregular deformations for the molded lenses in the sintering. Therefore, a constraint of $\frac{h}{d}\leq 0.2$ is purposely set for the height *h* and diameter *d* of the green body. After ejecting the green body, the solid‐state sintering with four stages, as presented in Figure 1c, is conducted to get the fully transparent fused silica optics.

**Figure 2:**
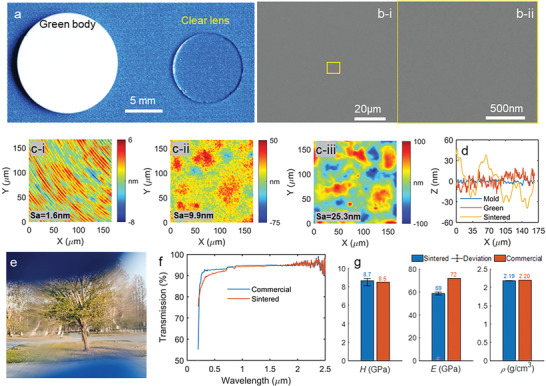
Basic features of a molded planar lens, a) the molded green compact and its sintered clear lens with obvious geometry shrinkages, b‐i) SEM images of the cross‐sectional surfaces of the dense glass, b‐ii) a close‐up view of an arbitrary spot in (b‐i), c‐i) the surface microtopography of the Ni‐P mold with clear tool marks and a roughness of *Sa* = 1.6 nm, c‐ii) the surface microtopography of the solidified green compact with a roughness of *Sa* = 9.9 nm, c‐iii) the surface microtopography of the dense glass with a roughness of *Sa* = 25.3 nm, d) the cross‐sectional profiles of the surface microtopography, e) a clear view of a tree through a sintered planar glass, f) the light transmittance over the UV–vis to the near infrared spectrum, g) a comparison between the sintered and commercial fused silica glass in terms of the hardness, Young's modulus, and density.

After molding with an axial pressure of ≈70 MPa, an opaque green body with a relative density of ≈36.5% was obtained, as photographically shown in **Figure** **2**a. In the sintering, the heating rate in the second stage (from *T*_1_ = 400 °C to *T*_2_ = 1300 °C) is set to be smaller than that used in the first heating stage to avoid possible cracks. Neck formation and boundary diffusion for the isolated pores happened between the contact nanopowders in the heating process, resulting in obvious shape shrinkages. The highest temperature *T*_2_ = 1300 °C was held for 10 min in the third stage to get the fully densified glass. After the natural cooling in the final stage, the fully transparent fused silica glass with shrinkage was obtained as shown in Figure 2a. A scanning electronic microscopic (SEM) image of the cross‐sectional surface of the sintered glass is illustrated in Figure 2b‐i, featuring a high uniformity without any defects. A close‐up view of an arbitrary spot on the cross‐sectional surface is further presented in Figure 2b‐ii, suggesting a fully densified structure without any pores, even at the nanoscale. The X‐ray diffraction (XRD) and Raman spectroscopy results for the sintered glass in Figure S1a,b (Supporting Information) show identical features to them of the commercial fused silica glasses, demonstrating the fully amorphous structure obtained by the room‐temperature molding. Furthermore, the transmission spectrum measured by a Fourier transform infrared spectroscopy (FTIR) also agreed well with the commercial fused silica glass (Figure S1c, Supporting Information), showing the high purity of the sintered glass without any undesired hydroxy groups.

In the sintering, the axial pressure and holding temperature are crucial factors controlling the derivation of the clear lenses. Usually, low pressing stress may lead to over‐large initial pores, which may remain in the sintering process due to the low capillary pressure of the pores. To investigate the effect of axial pressure on the glass molding, we pressed the nanopowders with pressures of around 40, 55, and 83 MPa at the room‐temperature to get the green bodies, which were then sent for the sintering using the consistent heating profile in Figure 1c. After the high‐temperature sintering, the resulting glasses in Figure S2a (Supporting Information) suggested that increasing the axial pressure may enhance the transparency. However, over‐large pressure (83 MPa, for example) may lead to undesired edge breakages and fractures since the shear stress in the ejection process might be larger than the green strength (Figure S2a, Supporting Information). After getting a set of green bodies using a consistent stress of ≈70 MPa, various holding temperatures were employed for different green bodies in the sintering to investigate the effect of the holding temperature on deriving the opaque lenses. From the photograph shown in Figure S2b (Supporting Information), a lower holding temperature of *T*_2_ = 1250 °C may result in the opaque body as observed in Figure S2b (Supporting Information), attributing to the insufficient sintering with unclosed pores as observed from the SEM images in Figure S2c (Supporting Information). By contrast, when a higher holding temperature is applied, the mitigation rate of powder boundaries might be faster than the pore coalescence rate. In this case (*T*_2_ = 1320 °C, for example), gas entrapments in the enclosed pores with further bloating may increase the porosity and lead to slight white defects (Figure S2d, Supporting Information). The enclosed pores at the nanoscale are clearly observed from the SEM images in Figure S2e (Supporting Information).

Assisted by the white light interferometry (WLI) ‐based optical surface profiler, the 3D surface microtopography of the components was captured to evaluate the surface roughness evolution in room‐temperature molding. For the molded component in Figure 2a, the surface topographies of the Ni‐P mold, green body, and sintered glass surfaces are illustrated in Figure 2c‐i–iii, respectively. Enabled by the ultraprecision diamond turning, high smoothness of the Ni‐P mold surface was obtained with a roughness of *Sa* = 1.6 nm. The surface of the pressed green body in Figure 2c‐ii shows small fluctuations with lateral sizes in tens of micrometers and amplitudes in tens of nanometers, resulting in a slightly larger surface roughness of *Sa* = 9.9 nm. The surface fluctuation might be caused by the remote nonuniform flow of the silica nanopowders at the interface between the mold and nanopowders. After the sintering, the fluctuations on the glass surface were enhanced owing to the nonuniform thermodynamic diffusions, leading to a slightly higher roughness of *Sa* = 25.3 nm in Figure 2c‐iii.

Through the sintered planar lens in Figure 2a, objects can be clearly observed, for example, a tree as observed through the lens in Figure 2e. To evaluate the optical performance of the sintered glass quantitatively, the transmittance was measured using the spectroscopy with the wavelength ranging from 250 to 2500 nm, resulting in the transmittance response in Figure 2f. Compared with the transmittance of a commercial fused silica glass, a nearly identical transmittance (≈95%) was obtained in the near‐infrared spectrum with the wavelength from 800 to 2500 nm, which went slightly smaller in the ultraviolet–visible spectrum (250 to 800 nm). However, over 90% transmittance was still obtained in the visible range. The decreased transmittance in the spectrum with shorter wavelengths might be mainly caused by the surface Rayleigh scattering, considering that the sample surface was polished to have a roughness of around *Sa* = 5 nm in the transmittance measurement. The refractive index of the sintered glass was measured by an ellipsometer with a wavelength ranging from 200 to 2000 nm. The obtained refractive index in Figure S1d (Supporting Information) exhibits a similar feature to that of a commercial fused silica glass. To evaluate the mechanical property of the sintered glass, three repetitive nanoindentation tests were conducted on various positions on the sample. The nearly identical indentation curves in Figure S1e (Supporting Information) suggested a highly uniform property of the sintered glass. From the derived properties summarized in Figure 2g, the micro‐hardness of the sintered glass was ≈*H* = 8.7 GPa, which is highly close to the reported hardness of commercial fused silica glasses (8.5 GPa). Furthermore, the elastic modulus of the sintered glass was around *E* = 59 GPa, which is ≈82% of the commercial glass. Through the immersion tests, the density of the sintered glass was ≈2.19 g cm^−3^, achieving a relative density of 99.55% concerning the theoretical density of 2.2 g cm^−3^ for the fused silica glass.

### 2.2 Molding a Miniature Spherical Lens

**Figure 3:**
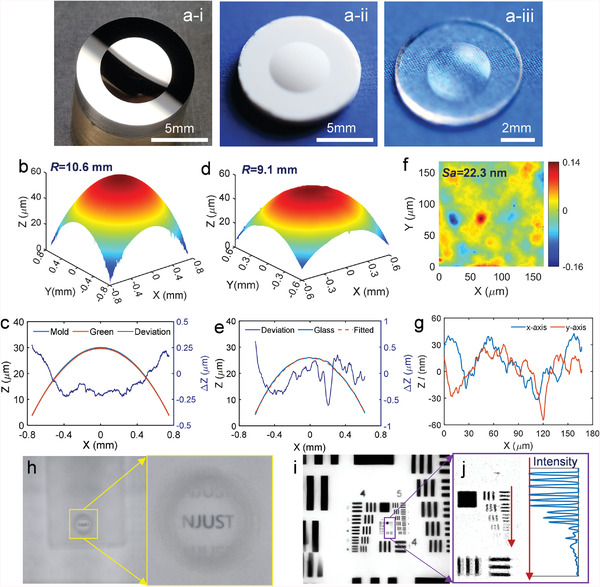
Results for molding a miniature spherical lens, a‐i) the Ni‐P mold with a concave spherical structure, a‐ii) the solidified green body, a‐iii) the sintered glass with full transparence, the 3D surface of b) the green body and d) the sintered glass measured by the WLI‐based optical surface profiler, which have equivalent curvatures of 10.6 and 9.1 mm, respectively. c) the cross‐sectional profiles of the mold and green body surface for the demonstration of the shape reservation capability, e) the cross‐sectional profiles of the sintered glass with its best‐fitted curve, f) the 3D roughness component by extracting the spherical substrate, g) cross‐sectional profiles of the surface roughness component, h) the captured image through the molded clear spherical lens with a close‐up view of the imaged letters “NJUST”, i) the captured image of a positive USAF1951 resolution target, and j) a close‐up view of the elements in the 7‐th group.

By the ultra‐precision diamond turning, a concave spherical shape was fabricated on the Ni‐P mold as photographically shown in **Figure** **3**a‐i, which had an aperture of 5 mm and a surface height of 0.3 mm. Using an axial pressure of ≈ 70 MPa, a green compaction body (Figure 3a‐ii) was successfully molded with a well‐replicated convex shape. After sintering using the heating profile, a fully transparent convex fused silica lens (Figure 3a‐iii) was obtained with shape shrinkages. Through the WLI‐based surface profiler, the 3‐D topography of the green body was measured and shown in Figure 3b. Considering the structure symmetry, only the cross‐sectional profiles of the mold and green body were extracted and comparatively shown in Figure 3c to demonstrate the shape replication capability. As shown in Figure 3c, within the measurement range having an arc height of ≈30 µm, a maximum peak‐to‐valley (PV) deviation between the two profiles was within 0.5 µm.

Furthermore, the 3D topography of the sintered lens is shown in Figure 3d. The sintered structure reserved the spherical shape with a best‐fitted radius of *R* = 9.1 mm, which was slightly smaller than that of the green body (*R* = 10.6 mm). Taking an average refractive index of 1.46 in the visible spectrum, the sintered lens may have a focus length of 17.435 mm and a numerical aperture of *NA* = 0.15. Considering the reserved spherical structure, it is intuitive that an identical planar shrinkage occurs during the sintering. The cross‐sectional profile of the sintered glass in Figure 3e is exhibited in good accordance with a best‐fitted spherical profile. The maximum deviation of ≈1 µm suggested a strong capability of reserving the molded shape in the sintering.

Using a 50× objective lens of the optical surface profiler, the surface micro‐topography within a small area of ≈167×167 µm^2^ was captured with a relatively high lateral resolution of 0.16 µm. By removing the low‐frequency surface form, the extracted surface roughness component in Figure 3f exhibits a mean square surface roughness of *Sa* = 22.3 nm. The cross‐sectional profiles of the roughness component in Figure 3g show a continuous shape varied within ±45 nm. Through the sintered lens, a sharp, magnified image of the letter “NJUST” was captured and illustrated in Figure 3h. To further investigate the optical characteristics, the imaging resolution of the lens was tested by imaging a positive USAF1951 resolution target, resulting in the image shown in Figure 3i. A close‐up view of the imaged target is presented in Figure 3j, together with the extracted intensity of the elements in the 7‐th group. The well‐identified 4‐th element in the 7‐th group suggested an imaging resolution of around 181 lp mm^−1^. By using the central wavelength of *λ_c_* = 550 nm for the white light illumination, the theoretical Rayleigh resolution limit could be estimated as $\frac{0.61\lambda_{c}}{NA}=\;2.24\;\mu m$, which was ≈ 223.5 lp mm^−1^. Although the practical resolution was close to the theoretical limit, there was much room for improvement, for instance, improving the surface smoothness, etc.

### 2.3 Molding Complex‐Shaped Lenses

**Figure 4:**
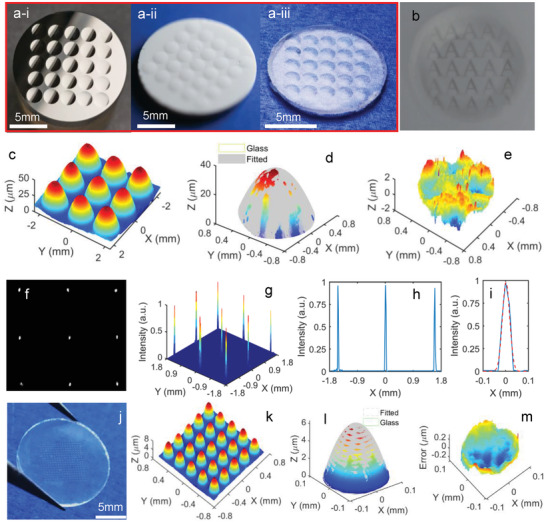
Results for molding a miniature lens array, a‐i) the Ni‐P mold with the arrayed concave structure, a‐ii) the solidified green body with the arrayed convex structure, a‐iii) the sintered lens array with full transparence, b) imaging of a letter “A” through the lens array, c) the 3D microtopography of the sintered lens array captured by the optical surface profiler, d) the extracted 3‐D topography with its best‐fitted sphere for an arbitrary lens in the lens array, e) the 3D deviation distribution of the sintered glass related to the best‐fitted sphere, f) the focused light spot through the lens array and g) its normalized light density distribution, h) the extracted cross‐sectional light density distribution for three sequential lenslets, i) the cross‐sectional density distribution for an arbitrary spot with the best‐fitted Gaussian curve, j) photograph of a molded micro‐lens array with much smaller feature sizes, k) the 3D micro‐topography captured by the optical surface profiler, l) a comparison between the best‐fitted and practical shape of an arbitrary spherical lenslet, and m) the corresponding form deviation distribution.

The practical molding of typical miniature lens arrays was conducted to demonstrate the versatility of the proposed room‐temperature molding method. Taking advantage of the ultra‐precision diamond turning, a typical miniature spherical lens array on the Ni‐P alloy was fabricated as the mold. The photography of the metallic mold, replicated green body, and sintered transparent glass is illustrated in **Figure** **4**a. The pitch, height, and aperture of the lenslets on the mold were designed as 150, 50, and 2 mm, respectively. By implementing the imaging system as illustrated in Figure S3 (Supporting Information), a uniform image of the letter “A” was observed through each lenslet as shown in Figure 4b. In addition, through the optical surface profiler, the 3D topography of the green body was measured and presented in Figure S4a (Supporting Information), showing uniformly distributed spherical structures. The cross‐sectional profile of an arbitrary lenslet in the green body was extracted against that of the mold in Figure S4b (Supporting Information). The maximum deviation without considering the boundary effect was smaller than 1 µm, exhibiting a good mold replication capability for the arrayed structure.

The 3D surface topography of the sintered glass was measured and presented in Figure 4c. To better characterize the lenslets, the 3‐D topography of an arbitrary lenslet was extracted and presented in comparison with a best‐fitted sphere in Figure 4d, leading to the extracted shape deviation within 3 µm as illustrated in Figure 4e. By replacing the letter “A” with the collimated light from a lamp, the focused light spot image captured through the lens array is shown in Figure 4f. By reverting the grayscale into the normalized intensity, the resultant light density distribution, as presented in Figure 4g, shows high uniformity and high sharpness. Accordingly, the cross‐sectional density distribution for three adjacent focused spots was extracted in Figure 4h. A separate view of one focused spot profile in Figure 4i with the best‐fitted Gaussian curve shows a full width at half maximum of around 38 µm (≈2.57% of the lenslet aperture).

Furthermore, a micro‐lens array was generated to demonstrate the feasibility of molding fused silica lens arrays with smaller feature sizes. The photograph of the molded micro‐lens array is shown in Figure 4j. The 3D micro‐topography captured by the optical surface profiler with a 5× objective lens is illustrated in Figure 4k, showing a highly uniform structure with an average height and aperture of 6.7 and 232.3 µm for the lenslet. After comparing the 3D shape of an arbitrary lenslet with the theoretical sphere in Figure 4l, the resulting deviation, as demonstrated in Figure 4m, was observed to be smaller than 0.48 µm (PV). Compared with the millimeter lenslet in Figure 4c, the smaller form error for the micro‐lenslet in Figure 4k had a favorable scale effect, suggesting that the proposed molding method may have better accuracy for producing smaller‐scale optics.

**Figure 5:**
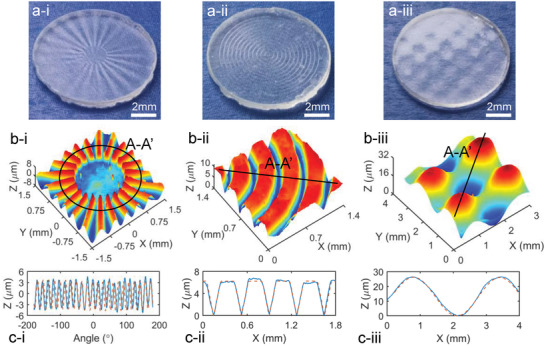
Results for molding optical lenses with various complex shapes, photography of a‐i) the affine array, a‐ii) the concentric micro‐grooved surfaces, and a‐iii) the micro‐grid freeform surface, the 3D microtopography measured by the optical surface profiler for b‐i) the affine array, b‐ii) the micro‐grooved surface, and b‐iii) the micro‐grid freeform surface, and the extracted cross‐sectional profiles along *A*‐*A’* for (c‐i) the affine array, c‐ii) the concentric micro‐grooved surfaces, and c‐iii) the micro‐grid freeform surface, where the blue and red lines represent profiles of the sintered glass and the shrank mold.

Various structured surfaces were further replicated to demonstrate the versatility of the proposed room‐temperature molding method, including the affine micro‐array, the concentric micro‐grooved surface, and the micro‐grid freeform surface. The resultant structured fused silica glasses with full transparency are photographically illustrated in **Figure** **5**a. Compared with the mold shapes in Figure S5 (Supporting Information), the desired surface shapes were replicated and reserved in the sintered glasses. By the optical surface profiler, the 3D topographies of the sintered glasses are shown in Figure 5b. The cross‐sectional profiles along the marked directions are further presented in Figure 5c, together with the shrank profiles from the molds. The continuously‐varying shapes and discontinuous surfaces with sharp edges in Figure 5b,c suggest the excellent capability for molding complicated surfaces with various features. By taking the micro‐grid freeform surface for instance, the best‐fitted 3D form with the shrinkage compensation was derived and comparatively shown in Figure S6a (Supporting Information), exhibiting a good agreement with the sintered glass surface. The deviation between the two surfaces was extracted to be ≈5.8 µm (PV), as shown in Figure S6b (Supporting Information).

### 2.4 Discussions

**Figure 6:**
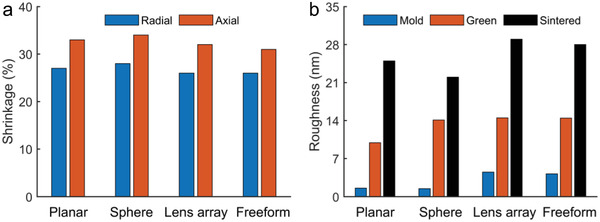
A summary of the shrinkage ratio and roughness for the planar, sphere, lens array and micro‐grid freeform surfaces, a) the radial and axial shrinkage ratios in the sintering, and b) the surface roughness for the mold, green body, and sintered glass.

To investigate the feature size and surface roughness evolution during the molding and sintering, we summarized the shrinkage ratios and surface roughness in **Figure** **6** for four typical surfaces, including the planar, spherical, lens array, and freeform surfaces. The axial pressure was manually controlled to be ≈70 MPa for the replication. After fitting surface shapes for the dense glasses, the directional shrinkages induced by the sintering were derived by comparing the feature sizes of the dense glass and the green body. The well‐reserved surface shape suggested an identical shrinkage along the radial direction for each case (≈ 26.7% on average), which is slightly smaller than that along the axial direction (32.5% on average) as shown in Figure 6a. This phenomenon might be caused by the fact that the nanopowder rearrangement in the axial pressing may lead to a denser structure along the radial direction. Notably, although the shrinkage tendency was similar for all the cases, the actual ratios were slightly different for all cases along both the radial and axial directions, owing to the non‐identical density of the compact green body induced by the non‐identical pressures in the molding.

Figure 6b summarizes the surface roughness evolution of the metal molds, green bodies, and sintered glasses. Taking advantage of the low system vibration and sharp tool edge in the ultra‐precision diamond turning, the resultant Ni‐P mold had an ultra‐high smoothness, i.e., *Sa*<5 nm for all the molds. In the axial pressing, the relatively strong surface forces normally led to poor mobility for the nanopowders, resulting in tiny fluctuations at the nanoscale as observed in Figure 2c,d. Therefore, the pressed green body had a slightly higher surface roughness of around *Sa* = 13 nm on average. After sintering, the powder repacking, surface diffusion, and structure shrinkages may magnify the surface defects induced by the non‐uniformity of the clustered nanopowders, leading to rougher glass surfaces with a roughness of around *Sa* = 26 nm on average. The essential thing for enhancing the surface smoothness might be the improvement of the free flow of the silica nanopowders by adopting proper mold coatings or vibration assistance. Furthermore, for applications requiring ultra‐smooth surfaces, ultra‐precision post‐processing polishing methods could be further employed to improve surface smoothness, for example, magnetorheological polishing, ion beam figuring, and plasma polishing, to mention a few.^[^ ^4^ ^]^

Another crucial factor determining potential applications of the molded lenses might be the form accuracy. For the lenses demonstrated in this study, the form errors (PV) ranged from hundreds of nanometers to several micrometers. The main factors causing such relatively large form errors might be the nonuniform structures in the green body and the nonuniform heating in the sintering. For achieving a high‐accuracy replication of fused silica lenses, the vibration assistance in the pressing might be promising for improving the structure uniformity. Furthermore, advanced heating techniques that can provide a more uniform heating profile inside the green body could effectively reduce the lens deformations in the sintering, for example, the spark plasma sintering.^[^ ^40^, ^41^ ^]^ Similar to the surface roughness improvement, the ultra‐precision deterministic polishing techniques would be powerful for further correcting surface form errors of sintered lenses downward to the nanometric level.

## 3 Conclusion

We proposed a new room‐temperature molding technique for the mass production of fused silica lenses with various complex shapes. By directly pressing the silica nanopowders against the metal mold in minutes, a consolidated silica green body without any additives was created with the replicated mold shape. After high‐temperature sintering in the air atmosphere, fully transparent fused silica glasses with well‐reserved shapes are derived. The proposed method demonstrates the flexible and efficient capabilities for mass reproducing high‐quality fused silica lenses with various shapes, including spherical, arrayed, and freeform patterns. This technique thus enables a new scalable, precise, and cost‐effective route for replicating fused silica lenses, potentially promoting numerous low‐cost applications for fused silica lenses in optics and photonics, as well as applications in chemical and biomedical chips.

## 4 Experimental Section

### Materials and Uniaxial Pressing

The slow tool servo‐based ultra‐precision diamond turning (MTC 350, LT Ultra, Germany) was employed to generate the optical mold with complex shapes. With the turning, the feedrate was set as 2 µm rev^−1^, and the spindle speed was adaptively determined according to the shape complexity to‐be‐turned. A natural diamond tool (Contour Fine Tooling, UK) with a nose radius of 1.111 mm was employed for the material removal. Brass samples with electroless nickel‐phosphorus (Ni‐P) alloy plating were adopted as the mold material for the lower die due to the relatively high hardness and high machinability of the Ni‐P material. Meanwhile, a planar die steel with a well‐polished surface was adopted as the upper die.

The silica nanopowders (Aerosil OX50, Evonik, Germany) with a nominal powder diameter of 40 nm and a purity higher than 99.8% were used as the raw material for the molding. The axial pressure provided by the hydraulic press was manually controlled to be ≈70 MPa, which dwelled for 1 min at room temperature. After the pressing, the silica nanopowders were solidified to the green compact.

### Heat Treatment

The obtained green body was sintered in the air atmosphere through a muffle furnace (Kejia, China). A two‐stage heating was employed by adopting a heating rate of 1 °C min^−1^ at the first stage (from room temperature to *T*_1_ = 400 °C) and a heating rate of 0.67 °C min^−1^ at the second stage (from *T*_1_ = 400 °C to *T*_2_ = 1300 °C) to avoid possible cracks. Before the natural cooling down to room temperature, the high‐temperature sintering at 1300 °C was held for 10 min to achieve a dense and transparent glass.

### Optical Performance Characterization

The transmittance feature from the ultraviolet to the near‐infrared spectrum was measured using a UV–Vis‐NIR Spectrometer (Shimadzu UV‐3600, Japan). To construct the imaging system presented in Figure S3 (Supporting Information), a CCD camera (SN‐60U2K, SangNond Corporation, China) was adopted for capturing images through the sintered fused silica optics, which had 1392×1024 pixels. After imaging the light spots, the greyscales of the captured images were extracted to represent the light intensity distributions as received.

### Surface Topography Characterization

Surface forms and roughness of the mold, green body, and sintered glass were measured by a white light interferometry‐based optical surface profiler (Newview 8200, Zygo Corporation, USA). To capture large‐area surface forms, a set of small areas with overlaps of ≈20% were measured using a 5× objective lens with a field of view (FOV) of ≈1.5×1.5 mm^2^ and a lateral resolution of 1.4 µm. After the measurement, the small areas were automatically stitched to obtain the large‐scale surface forms by the surface processing software from Zygo. Meanwhile, a 50× objective lens with a FOV of 0.16×0.16 mm^2^ and a lateral resolution of 0.16 µm was employed to obtain surface roughness components, which were extracted by removing the surface forms. For observing the internal structures of the sintered glasses, the cross‐sectional surfaces obtained by breaking the glass were measured by an ultra‐high resolution scanning electron microscope (ZEISS Sigma 300, Germany).

### Glass Property Characterization

To identify the material components in the sintered glass, the transmission over a broad infrared spectrum was measured using Fourier transform infrared spectroscopy (Thermo Scientific Nicolet iS5, USA). Raman spectroscopy was obtained by Raman microscopy (Horiba LabRAM HR Evolution, Japan) with a laser wavelength of 532 nm. To characterize the crystalline feature of the sintered glass, the X‐ray diffraction testing was implemented by an X‐ray diffractometer (Bruker D2 Phaser, Germany) with a scan rate of 5°min^−1^. The density of the sintered glass was measured by the Archimedes method at room temperature (23 °C). To identify the micro‐hardness and Young's modulus of the glass, the nanoindentation was conducted by a nanoindentation instrument (Bruker Hysitron TI980, Germany). For the indentation, the maximum load was set as 2 mN, and the loading and unloading rate was set as 400 µN s^−1^. The measurement of the refractive index was performed on an ellipsometer (Horoba UVISEL PLUS, France).

## Conflict of Interest

The authors declare no conflict of interests.

## Supporting information

Supporting Information

## Data Availability

The data that support the findings of this study are available from the corresponding author upon reasonable request.;
