# Supplementary material for: Room‐Temperature Molding of Complex‐Shaped Transparent Fused Silica Lenses
Source: Adv Sci (Weinh). 2023 Oct 23;10(34):2304756. doi: 10.1002/advs.202304756 (PMC10700191; doi:10.1002/advs.202304756)
Supplement: Supplementary file 1 — Supporting Information [file ADVS-10-2304756-s001.pdf]

ADVANCED SCIENCE
Open Access

Supporting Information

for *Adv. Sci.*, DOI 10.1002/advs.202304756

Room-Temperature Molding of Complex-Shaped Transparent Fused Silica Lenses

*Ya Xu, Xiaotong Du, Zhenhua Wang, Hua Liu, Peng Huang, Suet To, LiMin Zhu and Zhiwei Zhu**

Supporting Information

Room-temperature molding of complex-shaped transparent fused silica lenses

*Ya Xu, Xiaotong Du, Zhenhua Wang, Hua Liu, Peng Huang, Suet To, LiMin Zhu, and Zhiwei Zhu**

Wall friction-induced pressure gradient

The pressure decreases gradually from the upper punch to the bottom of the compact owing to the friction between the nanopowders and the side wall, which may influence the compact density distribution along the height direction. Assuming the pressure at the upper punch is P_0, the pressure $P(z)$ and the relative density $\rho_r(z)$ over the axial motion z can be described as [1],[2]

$$P(z)=P_0\exp\left(-4\mu k\frac{z}{d}\right) \# \text{(S1)}$$

$$\ln\left(\frac{1}{1-\rho_r(z)}\right)=a+cP_{up}\exp\left(\ln\left(\frac{P_{low}}{P_{up}}\right)\frac{z}{h}\right) \# \text{(S2)}$$

where μ is the friction coefficient, k is the radial pressure coefficient, ρ_r is the relative density of the compact, and d and h are the diameter and height of the compact, respectively. In addition, P_{low} and P_{up} are pressures on the lower and upper surface of the compact, and a and c are the constants. According to the Equations S1 and S2, increased pressure and density differences between the bottom and top of the compact may occur with the increase of the height to diameter ratio. The increased structure nonuniformity may lead to heavier warpage and irregular deformations of the lenses in the sintering process. Thus, a sufficiently small height to diameter ratio was required to guarantee a relative homogeneous pressure and density distribution in the compact.

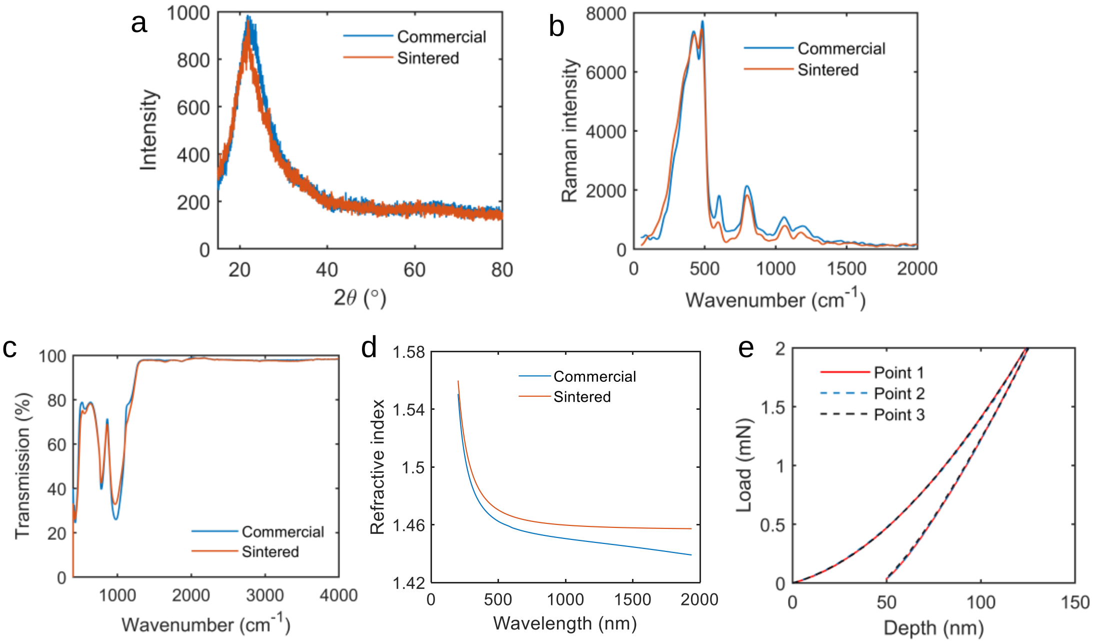

Figure S1. Characterization of the sintered glass, (a) X-ray diffraction result of the sintered glass and commercial fused silica glass, (b) Raman spectrum of the sintered and commercial fused silica glass, (c) Fourier transform infrared results of the sintered and commercial fused silica glass, (d) refractive index of the sintered and commercial fused silica glass, and (e) load-displacement curves measured by the nanoindentation at three arbitrary points on the sintered glass.

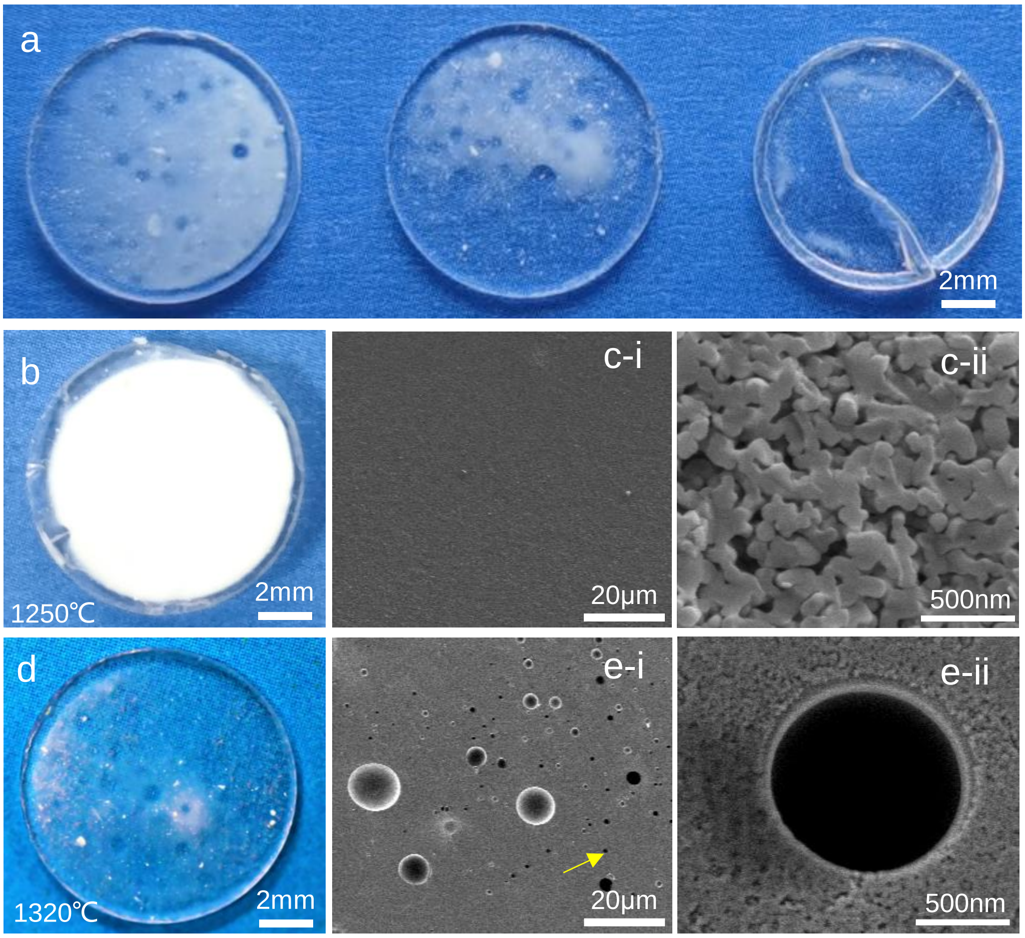

Figure S2. Results of the densified glasses under various conditions, (a) photograph of the sintered glasses which were derived from green bodies obtained under various axial pressures of 40 MPa, 55 MPa and 83 MPa at the room-temperature, (b) photograph of the sintered glass with a dwell temperature of 1250 °C, (c-i) SEM image of the cross-sectional surface of the component in (b) and (c-ii) its close-up view, (d) photograph of the sintered glass with a dwell temperature of 1320 °C, and (e-i) SEM image of the cross-sectional surface of the component in (d) and (e-ii) its close-up view.

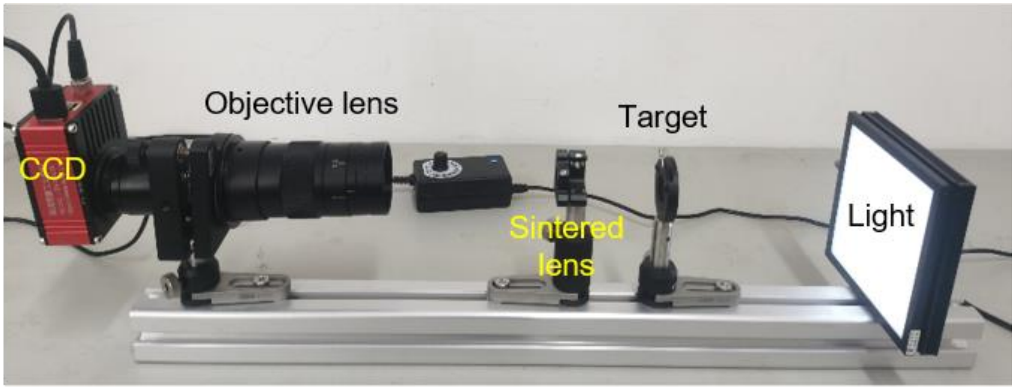

Figure S3. Photograph of the experiment setup for the imaging, which contains a CCD camera with a set of objective lenses with a magnification ratio of 5× and a diffused light source.

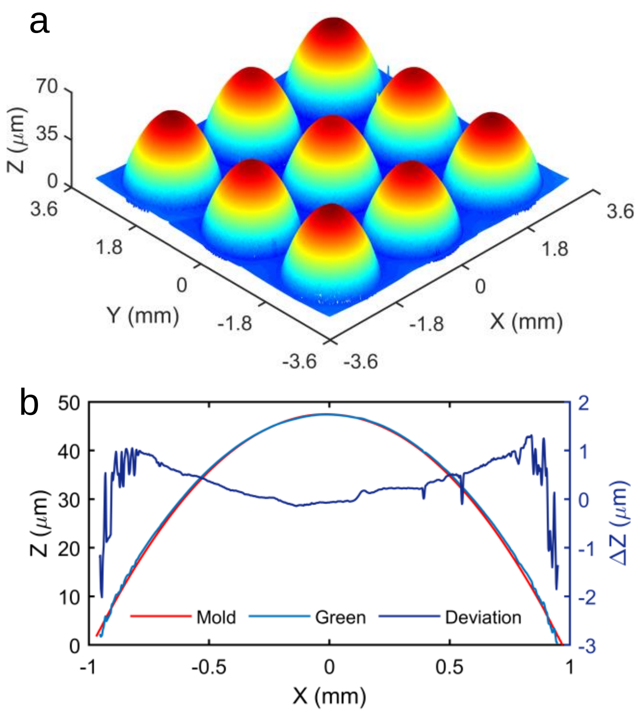

Figure S4. Features of the lens array, (a) 3-D topography of the molded green body of the lens array, and (b) the cross-sectional profile for one lenslet together with its profile deviation.

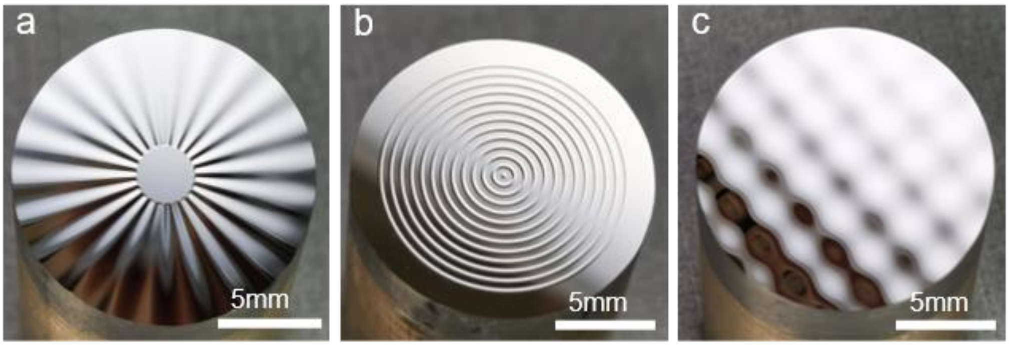

Figure S5. Ni-P molds with various shapes, (a) affine array, (b) concentric micro-grooved surface with sharp edges, and (c) micro-grid freeform surface.

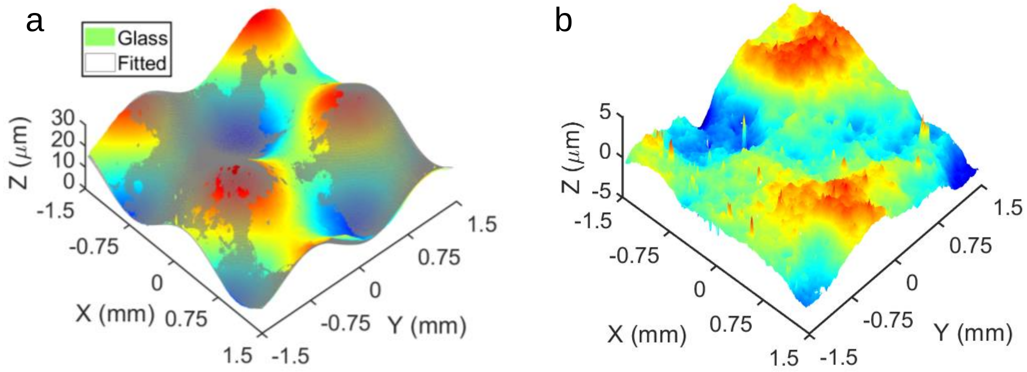

Figure S6. Characteristics of the micro-grid freeform surface, (a) the extracted 3-D topography with its fitted surface, and (b) the form deviation distribution between the sintered glass and its best-fitted surface with shrinkage compensation.

Reference

[1] R. Ralf, and I-W. Chen, eds. **2011**, John Wiley & Sons.

[2] MS. Kadiri, A. Michrafy, JA. Dodds, *Powder Technol* **2005**, *157*, 1-3.
